# Strawberry micropropagation simplifies endomicrobiome and highlights potential for *in vitro* biotization with beneficial rhizobacteria

**DOI:** 10.1111/plb.70278

**Published:** 2026-08-16

**Authors:** A. M. G. Sepúlveda, E. da Conceição Jesus, Y. C. Molina, V. H. Buttrós, L. Q. Carvalho, M. Pasqual, J. Dória

**Affiliations:** ^1^ Department of Biology Federal University of Lavras Lavras Brazil; ^2^ Facultad de Ciencias Naturales, Agro‐Environmental Biotechnology and Health Universidad de Santander Bucaramanga Colombia; ^3^ Embrapa Agrobiologia Rio de Janeiro Brazil; ^4^ Department of Biology University of the Andes Mérida Venezuela; ^5^ Soil Department Federal University of Rio Grande do Sul Porto Alegre Brazil; ^6^ Department of Agriculture Federal University of Lavras Lavras Brazil

**Keywords:** *Azospirillum brasilense*, *Bacillus subtilis*, endophytes, flavonoids, phenolics

## Abstract

- Strawberry micropropagation produces uniform, disease‐free planting material. However, repeated *in vitro* subculturing can simplify the endophytic microbiome, reducing plant vigour and acclimatization success. This study investigated the impact of successive *in vitro* generations on the endomicrobiome of *Fragaria* × *ananassa* cv. San Andreas and determined if targeted biotization with beneficial rhizobacteria can enhance plant performance.
- Profiled endophytic bacterial and fungal communities were analysed in the mother plant and across three *in vitro* generations using amplicon sequencing of the 16S rRNA gene and ITS markers. Ten plant growth‐promoting bacterial strains were tested *in vitro*. The most effective strains were combined into consortia, and their impact on growth, survival and phenolic and flavonoid accumulation was assessed under *in vitro* and *ex vitro* acclimatization conditions.
- Successive subcultures simplified the endophytic assemblage. The mother plant exhibited substantially higher bacterial alpha‐diversity than *in vitro* generations, with richness and Shannon diversity declining significantly (*P* <0.001) across all subcultures. *In vitro* biotization with *A. brasilense* Ab‐V5 and *A. brasilense Ab‐V6* increased total dry biomass from 190.63 mg in the control to 216.75 mg and 209.11 mg, respectively. Under *ex vitro* conditions, several inoculated treatments achieved 100% survival compared with 75% in the non‐inoculated control.
- Prolonged *in vitro* maintenance reduces the complexity of the strawberry endomicrobiome by imposing a selective bottleneck. Targeted introduction of beneficial bacteria partially compensates by enhancing growth, survival and secondary metabolite accumulation, supporting microbiome‐assisted strategies to improve tissue culture performance and *ex vitro* establishment.

## Graphical abstract

Prolonged *in vitro* micropropagation reshaped the strawberry endomicrobiome, reducing bacterial diversity and community complexity. Biotization with selected plant growth‐promoting bacteria partially compensated for these changes by improving plant performance and acclimatization, supporting microbiome‐assisted approaches for tissue culture systems.

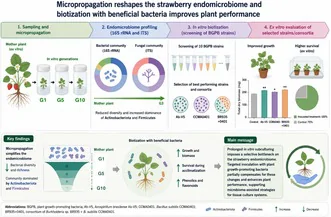

## INTRODUCTION

Strawberry (Fragaria × ananassa Duch.) is an economically important crop, widely cultivated because of its nutritional value and high content of bioactive compounds, including vitamin C, phenols, flavonoids, anthocyanins and tannins, which are associated with health‐promoting properties (Fierascu *et al*. 2020; Mezzetti *et al*. 2018). High‐quality planting material is crucial for sustainable strawberry production, and *in vitro* micropropagation has become a key strategy for generating large numbers of genetically uniform and pathogen‐free plantlets. However, repeated subculturing in artificial and aseptic conditions may alter plant‐associated microbial communities, affecting plant vigour and acclimatization (Read & Preece 2014; Soumare *et al*. 2021; Abdalla *et al*. 2022). Although the importance of plant‐associated microbiomes for plant performance is increasingly recognized (Luo *et al*. 2025), little is known about how successive cycles of strawberry micropropagation influence endophytic microbial diversity and whether the resulting changes can be mitigated through targeted microbial interventions.

The success of *in vitro* culture depends on several factors, including explant type, culture medium, sources and concentrations of sugars, and growth regulators, among others. These factors directly influence plant development, affecting leaf size, microplant height, root development, callus or shoot formation and multiplication rate (Murashige & Skoog 1962; Grattapaglia & Machado 1998; Golle *et al*. 2012). Additionally, issues such as oxidation and contamination can negatively impact *in vitro* culture. Any change in these parameters may alter plant development and affect acclimatization success (Bekalu *et al*. 2023). During *in vitro* establishment, explants undergo surface sterilization to eliminate epiphytic microorganisms; however, endophytic microorganisms that naturally inhabit internal plant tissues survive the sterilization procedures used in micropropagation (Orlikowska *et al*. 2016; Quambusch & Winkelmann 2018). Beneficial endophytes colonize internal plant tissues without causing disease and can contribute to nutrient acquisition, modulation of plant metabolism, protection against pathogens and increased resilience under adverse conditions (Hardoim *et al*. 2015; Ali *et al*. 2024). Consequently, alterations in endophytic communities during micropropagation may have important implications for the subsequent development and acclimatization of micropropagated plants (Xiang *et al*. 2023; Vinskienė *et al*. 2025). Such alterations in endophytic microorganisms are not solely a result of surface sterilization but also stem from the selective pressures exerted by *in vitro* conditions. These conditions include artificial nutrient media, elevated humidity, restricted gas exchange and diminished microbial immigration from rhizosphere to endophytes (Frank *et al*. 2017; Mushtaq *et al*. 2023). These factors may selectively favour a subset of endophytic microorganisms.

The effects of endophytes in *in vitro* culture depend on factors such as plant genotype, culture conditions and the identity of the associated microorganisms (Fouda *et al*. 2019; Fouda *et al*. 2021). Nevertheless, evidence points that endophytic microorganisms can contribute to the development and acclimatization of micropropagated plants by influencing growth, nutrient acquisition and stress responses (Luti & Mavituna 2010; Maheswari *et al*. 2013; Song *et al*. 2017; Fouda *et al*. 2021; Jakupi & Demirbas 2022). Understanding the diversity and dynamics of endophytic communities associated with explants is therefore essential, and endophytic communities have already been described in other plant species such as *Pogonatherum paniceum* (Koskimäki *et al*. 2010a; Koskimäki *et al*. 2010b), *Atriplex* spp. (Lucero *et al*. 2011), *Prunus avium* (Quambusch & Winkelmann 2018), *Arbutus unedo* L. (Martins *et al*. 2022), demonstrating that *in vitro* culture can reduce endophytic diversity and reshape microbial assemblages, potentially influencing plant development and acclimatization. Despite the growing interest in endophytic microbial communities, the temporal dynamics of bacterial and fungal endophytes during successive cycles of strawberry micropropagation remain less understood.

Because *in vitro* micropropagation may simplify the endophytic microbiome, it also provides a controlled platform for testing microbiome‐assisted strategies such as biotization with plant growth‐promoting bacteria (PGPB). Biotization involves inoculating plant tissue explants or microplants under *in vitro* conditions with beneficial microorganisms, thereby enabling the study of metabolic responses and plant development (del Rosario Espinoza‐Mellado *et al*. 2021). In addition to promoting plant growth, biotization may partially compensate for the functional simplification associated with prolonged *in vitro* culture and improve plant performance during acclimatization (Vinskienė *et al*. 2025). Successful applications of biotization have also been reported in other horticultural crops, including apple and banana, where inoculation with beneficial microorganisms improved rooting, growth and acclimatization performance (Xiang *et al*. 2023; Vinskienė *et al*. 2025). In this research, we investigated the endophytic bacterial and fungal communities associated with strawberry microplants of the San Andreas cultivar across successive *in vitro* generations. We also evaluated the impact of plant growth‐promoting bacteria (PGPB) on plant growth, survival and secondary metabolite accumulation under both *in vitro* and *ex vitro* conditions. Our hypothesis was that microbial diversity would decrease across generations, negatively affecting plant performance. We hypothesized that targeted inoculation with beneficial bacteria could partially mitigate these effects by enhancing plant performance.

## MATERIAL AND METHODS

All experiments were conducted in three independent experimental runs under the same conditions, and the results presented are representative of these independent experiments.

### Microplant source and propagation

Stolons of the San Andreas (SA) cultivar, obtained from healthy mother plants, were provided by the Olericulture Laboratory of the Department of Agriculture at UFLA. For *in vitro* establishment, selected stolons were first washed and kept in sterile distilled water for 30 min under magnetic stirring. They were then surface sterilized with 70% ethanol for 2 min, followed by immersion in 1% sodium hypochlorite for 10 min, and subsequently rinsed six times with sterile distilled water. After this process, stolons were dried with sterile paper towels under aseptic conditions inside a laminar flow chamber (Tinak Ekom *et al*. 2026).

For micropropagation, stolons were transferred to 200 mL flasks containing 50 mL of basal MS medium (Murashige & Skoog 1962), supplemented with 1 mg l^−1^ 6‐benzylaminopurine (BAP), according to the protocol adapted from Boxus (1999). The medium pH was adjusted to 5.7 before autoclaving at 121 °C and 15 psi for 20 min. Cultures were maintained for approximately 30 ± 3 days in a growth room under controlled conditions of artificial light and temperature. Light was provided by white LED lamps (6400 K, λ = 525 nm) with an average irradiance of 49.4 μMol m^−2^ s^−1^ and a 16 h photoperiod. Temperature was maintained at 26 °C ± 2 °C (Araújo *et al*. 2021; Araújo *et al*. 2022).

### DNA extraction for amplicon sequencing

*In vitro* plantlets of the SA cultivar were subcultured every 30 days for 10 months. Plant material from microplants of three generations (first, fifth and tenth months, respectively, G1, G5, G10), as well as from the mother plant (MP), was grinded in liquid nitrogen for total DNA extraction using the DNeasy® Plant Mini Kit (Qiagen), according to the manufacturer's instructions. The quality and quantity of the extracted DNA were assessed by 1% agarose gel electrophoresis and spectrophotometry on a NanoDrop Lite (ThermoFisher Scientific) (260/280 nm). For each generation, 20 plantlets were sampled and distributed into four biological replicates, each consisting of five independent plants. DNA extraction was performed separately for each biological replicate, ensuring that the replicates represented independent biological samples rather than technical replicates.

### PCR and sequencing

PCR amplification and sequencing were carried out by BPI Biotecnologia EPP (São Paulo, Brazil). For bacterial community profiling, primers targeting the V5–V7 region of the 16S rRNA gene were used (Chelius & Triplett 2001; Beckers *et al*. 2016; Qiu *et al*. 2020). For fungal community profiling, the ITS1F/ITS2 primer set was applied, a standard of the Earth Microbiome Project for ITS metabarcoding (Gardes & Bruns 2008; Ihrmark *et al*. 2012; Bokulich & Mills 2013; Hoggard *et al*. 2018). Detailed primer information is provided in Table S1.

PCR conditions for 16S V5–V7 were an initial denaturation at 94 °C for 3 min, followed by 30 cycles of 94 °C for 45 s, 55 °C for 1 min, 72 °C for 1 min and a final extension at 72 °C for 10 min. Products (~800 bp and ~600 bp) were visualized on 1% agarose gels stained with UniSafe Dye (0.03% v/v), and the ~600 bp band corresponding to 16S was excised and purified with Agencourt AMPure XP magnetic beads (Beckman Coulter). For ITS1F/ITS2, PCR was performed with an initial denaturation at 95 °C for 3 min, followed by 35 cycles of 95 °C for 30 s, 55 °C for 30 s, 72 °C for 30 s and a final extension at 72 °C for 5 min, resulting in amplicons of ~300–400 bp depending on taxa. Indexing was performed with the Nextera XT Index Kit (Illumina), followed by purification with AMPure XP beads and quantification using the KAPA Library Quantification Kit (Roche). Equimolar pools of libraries were generated and sequenced on an Illumina MiSeq platform (MiSeq V2 Nano, 2 × 250 bp paired‐end) with a 25% PhiX spike‐in. All samples are accessible under the BioProject ID PRJNA1417270.

### Bioinformatics and sequencing data analysis

The sequences were analysed using the DADA2 pipeline (version 1.8) (Callahan *et al*. 2016) in R 4.5.2, where they were demultiplexed and quality filtered. For 16S, low‐quality sequences and chimeras (<2%) were removed, and all retained sequences had a quality score ≥Q30; forward reads were truncated at 240 bp, and reverse reads at 160 bp. For ITS, primer sequences were first removed with *Cutadapt* and reads shorter than 50 bp were discarded. After filtering, an average of 11,122 high‐quality sequences per sample was obtained across datasets. Amplicon sequence variants (ASVs) were inferred with DADA2, and taxonomic affiliation was performed using the SILVA v.138.2 database for 16S rRNA gene sequences (Quast *et al*. 2013) and the UNITE database (dynamic release 19.02.2025) for ITS sequences. Downstream analyses were conducted with the phyloseq package (McMurdie & Holmes 2013).

Microbial community composition at the phylum (16S) and genus (ITS) levels was summarized by relative abundance bar plots, with taxa representing ≤1% of reads in each sample grouped as ‘Other’. Beta diversity was assessed using non‐metric multidimensional scaling (NMDS) based on Bray–Curtis dissimilarities of square‐root–transformed ASV counts. Group differences in community structure were tested by PERMANOVA with 999 permutations (adonis2, vegan), reporting F and *P* values (Oksanen *et al*. 2015). Alpha‐diversity was quantified using observed ASVs, Shannon, Simpson and Pielou's evenness indices. Between‐group differences in alpha metrics were evaluated with parametric or pairwise tests, with Holm's adjustment for multiple comparisons (Patil 2021). Heatmaps of the 20 most abundant genera were constructed to visualize relative abundances (Liu *et al*. 2021).

### *In vitro* Biotization of San Andreas strawberry explants

Initially, 10 bacterial strains were used for the individual biotization of the SA cultivar, which were provided by the Culture Collection of Agricultural Microbiology (CCMA/UFLA) of the Laboratory of Fermentation Microbiology, Department of Biology, Federal University of Lavras (UFLA) and by the Johanna Döbereiner Biological Resource Center of Embrapa Agrobiologia, Brazil (CCBD), being: *Azospirillum brasilense* Ab‐V5, *Azospirillum brasilense* Ab‐V6, *Rhizobium tropici* CIAT 899, *Rhizobium leucaneae* BR935, *Sinorhizobium fredii* BR921, *Sinorhizobium fredii* CCMA0122, *Bradyrhizobium japonicum* CCMA 0088, *Bacillus subtilis* CCMA0401, *Bacillus megaterium* CCMA0004 and *Bacillus amyloliquefaciens* CCMA0112 (Table S2).

For biotization, explants measuring 2–2.5 cm in length, with 6–7 leaves and roots 3–5 mm in length, were selected. These explants were individually transferred to 200 mL flasks containing 50 mL of Murashige & Skoog (MS) basal medium (Murashige & Skoog 1962) and maintained for approximately 30 ± 3 days in a growth room under artificial light provided by white LED lamps (6400 K–λ = 525 nm), with an average irradiance of 49.4 μMol m^2^ s^−1^, a 16 h daily photoperiod and controlled temperature of 25 °C ± 2 °C (Araújo *et al*. 2021). After 1 week of acclimatization in the growth room, the explants were biotized in a laminar flow hood with 50 μL of a previously prepared bacterial suspension at approximately 1.5 × 10^8^ CFU/mL. The suspension of each treatment was applied at the stem–root base of each microplant, resulting in a final bacterial concentration of approximately 10^6^ cells per microplant. A completely randomized design was used with 10 treatments, each consisting of 24 plantlets (six per replicate, with four replicates per treatment).

Individual biotization allowed the selection of the four best‐performing strains, namely *Azospirillum brasilense* (Ab‐V5 and Ab‐V6), *Bacillus subtilis* CCMA0401 and *Rhizobium leucaneae* BR935, which were subsequently used to form consortia from their combinations (Table S2). The bacterial consortia were prepared by mixing individual bacterial suspensions in a 1:1 ratio and agitating at 100 rpm for 20 min at room temperature to ensure homogenization (Blanco et al. 2021).

### Acclimatization and *ex vitro* conditioning

After biotization with consortia, the treatments were established for transfer to *ex vitro* conditions, using the four individual strains and the five formed consortia (Table 1, Section 3). A completely randomized design was used with 10 treatments (4 strains and 5 consortia) and a control without inoculation, with 4 replicates. Each treatment consisted of 24 plantlets (six plantlets per replicate).

**Table 1:** Phytotechnical variables in San Andreas strawberry plants.

	shoot length	root length	leaf diameter	shoot dry mass	root dry mass	total dry mass
Section 1–Individual Strains *in vitro*						
*A. brasilense ab‐V5*	3.90 ± 0.01a	3.00 ± 0.01a	‐	175.25 ± 0.20a	41.50 ± 0.30a	216.75 ± 0.30a
*A. brasilense ab‐V6*	3.73 ± 0.08ab	2.93 ± 0.02a	‐	169.88 ± 0.40ab	39.23 ± 0.90a	209.11 ± 1.10a
*R. tropici cIaT 899*	2.78 ± 0.03e	1.00 ± 0.00e	‐	81.25 ± 1.30c	20.13 ± 0.20e	101.38 ± 1.30d
*R. leucaneae bR935*	3.50 ± 0.06bc	1.61 ± 0.04bc	‐	164.63 ± 0.20ab	31.00 ± 0.10b	195.63 ± 0.20bc
*S. fredii bR921*	3.03 ± 0.08de	1.33 ± 0.02d	‐	164.00 ± 0.30ab	24.88 ± 0.20cd	188.80 ± 0.20c
*S. fredii ccMa 0122*	0.00 ± 0.00 g	0.00 ± 0.00 g	‐	8.12 ± 0.09e	4.83 ± 0.10d	12.95 ± 0.10f
*B. japonicum ccMa 0088*	1.78 ± 0.03f	0.45 ± 0.01f	‐	17.25 ± 0.10e	4.21 ± 0.30e	21.46 ± 0.30f
*B. subtilis ccMa 0401*	3.88 ± 0.05a	1.80 ± 0.06b	‐	167.11 ± 1.40ab	37.75 ± 0.20a	204.86 ± 1.50ab
*B. megaterium ccMa 0004*	1.90 ± 0.03f	0.66 ± 0.04f	‐	41.68 ± 1.00d	7.54 ± 0.40e	49.20 ± 1.20e
Control	3.24 ± 0.02cd	1.49 ± 0.02cd	‐	163.25 ± 1.20b	27.38 ± 0.10bc	190.63 ± 1.20c
Section 2–Bacterial consortia						
ab‐V5 + bR935	5.98 ± 0.03a	1.72 ± 0.03b	‐	214.17 ± 0.90c	62.80 ± 0.40b	277.00 ± 1.30a
ab‐V5 + 0401	4.70 ± 0.02b	2.40 ± 0.02a	‐	233.30 ± 0.20b	63.67 ± 0.40b	297.00 ± 0.50a
bR935 + 0401	2.77 ± 0.02d	1.30 ± 0.01c	‐	148.50 ± 0.30e	40.83 ± 0.20c	189.33 ± 0.50b
ab‐V6 + bR935	3.35 ± 0.01c	1.55 ± 0.01bc	‐	150.00 ± 0.30e	42.60 ± 0.10c	192.60 ± 0.30b
ab‐V6 + 0401	5.88 ± 0.04a	2.12 ± 0.01a	‐	264.80 ± 1.40a	99.60 ± 0.20a	364.40 ± 1.60a
control	3.30 ± 0.01c	1.51 ± 0.07bc	‐	164.83 ± 0.30d	28.17 ± 0.10d	193.00 ± 0.20b
Section 3–Biotization (A)–after acclimatization						
*Azospirillum brasilense* Ab‐V5	12.13 ± 0.2a	15.54 ± 0.24d	14.16 ± 0.13a	1.17 ± 0.05ab	0.83 ± 0.06ab	2.00 ± 0.09a
*Azospirillum brasilense* Ab‐V6	12.46 ± 0.07a	17.25 ± 0.21c	13.40 ± 0.10ab	1.06 ± 0.06b	0.41 ± 0.04de	1.47 ± 0.08cd
*Rhizobium leucaneae* BR935	10.69 ± 0.15b	10.71 ± 0.14e	8.64 ± 0.30e	0.99 ± 0.07bc	0.49 ± 0.04cde	1.48 ± 0.08cd
*Bacillus subtilis* CCMA 0401	12.29 ± 0.17a	15.39 ± 0.10d	14.26 ± 0.08a	1.34 ± 0.03a	0.67 ± 0.06bc	2.01 ± 0.06a
Ab‐V5 + BR935	9.20 ± 0.20c	18.50 ± 0.21c	10.20 ± 0.26d	0.74 ± 0.05cde	0.90 ± 0.06a	1.64 ± 0.10bc
Ab‐V5 + 0401	10.15 ± 0.16bc	28.19 ± 0.35a	11.00 ± 0.19cd	0.72 ± 0.05de	0.50 ± 0.03cde	1.22 ± 0.06de
BR935 + 0401	12.47 ± 0.12a	21.78 ± 0.33b	13.13 ± 0.27b	1.20 ± 0.06ab	0.70 ± 0.03abc	1.90 ± 0.05ab
Ab‐V6 + BR935	7.85 ± 0.30d	6.81 ± 0.40f	7.28 ± 0.27f	0.24 ± 0.02f	0.18 ± 0.01f	0.42 ± 0.02f
Ab‐V6 + 0401	9.98 ± 0.18bc	15.96 ± 0.22d	10.49 ± 0.17d	0.53 ± 0.03e	0.36 ± 0.04ef	0.89 ± 0.07e
Control (A)	11.86 ± 0.20a	15.54 ± 0.31d	11.75 ± 0.23c	0.95 ± 0.07bcd	0.60 ± 0.04cd	1.55 ± 0.09bcd
Section 4–Biotization + Reinoculation (B)–after acclimatization						
*Azospirillum brasilense* Ab‐V5	11.01 ± 0.18cd	15.85 ± 0.18c	12.54 ± 0.12bc	1.39 ± 0.05b	1.37 ± 0.06a	2.76 ± 0.03a
*Azospirillum brasilense* Ab‐V6	11.66 ± 0.20bc	18.58 ± 0.30b	10.28 ± 0.17e	0.40 ± 0.03d	0.29 ± 0.02de	0.69 ± 0.03e
*Rhizobium leucaneae* BR935	10.74 ± 0.17d	9.79 ± 0.19e	11.41 ± 0.19d	0.86 ± 0.05c	0.53 ± 0.08c	1.39 ± 0.05cd
*Bacillus subtilis* CCMA 0401	10.69 ± 0.18d	12.90 ± 0.23d	12.05 ± 0.1cd	0.87 ± 0.04c	0.45 ± 0.03cd	1.32 ± 0.05d
Ab‐V5 + BR935	12.15 ± 0.08b	22.74 ± 0.17a	13.30 ± 0.30ab	1.40 ± 0.05b	0.56 ± 0.03c	1.96 ± 0.06b
Ab‐V5 + 0401	13.22 ± 0.09a	23.31 ± 0.70a	14.03 ± 0.26a	1.86 ± 0.08a	1.01 ± 0.05b	2.87 ± 0.10a
BR935 + 0401	11.04 ± 0.20cd	19.84 ± 0.25b	9.59 ± 0.17e	1.00 ± 0.03c	0.63 ± 0.04c	1.63 ± 0.07c
Ab‐V6 + BR935	5.28 ± 0.19e	6.44 ± 0.23f	5.29 ± 0.32f	0.20 ± 0.06d	0.17 ± 0.06e	0.37 ± 0.01f
Ab‐V6 + 0401	5.19 ± 0.17e	7.19 ± 0.08f	4.88 ± 0.22f	0.39 ± 0.02d	0.24 ± 0.02e	0.63 ± 0.03ef
Control (B)	12.05 ± 0.20b	15.23 ± 0.19c	11.45 ± 0.27d	0.91 ± 0.06c	0.61 ± 0.04c	1.52 ± 0.02cd

*Note*: (mean ± SE) | Shoot length (cm), root length (cm), shoot dry mass (mg), root dry mass (mg) and total dry mass (mg). Different letters indicate statistically significant differences (*P* < 0.05).

After evaluation of the phytotechnical variables, carried out 30 ± 3 days after biotization, the seedlings were transplanted into pots containing a previously sterilized mixture of the commercial substrate ‘Tropstrato® HT Hortaliças’ and sand in a 3:2 ratio. Sterilization was performed by autoclaving the mixture three times at 121 °C for 1 h. The seedlings were transferred to a greenhouse with controlled temperatures of 25 °C ± 2 °C during the day and 16 °C ± 2 °C at night. To facilitate adaptation to the new environment, the plants were covered with perforated polypropylene plastic, remaining under this protection for 2 h every 24 h during the first 4 days (96 h).

The transplanted seedlings were divided into two groups: the first group consisted of seedlings that were only biotized (A), while the second group received a second reinoculation (B). The seedlings were irrigated every 2 days, and the treatments were fertilized weekly with 10 mL of half‐strength Hoagland solution (Jarstfer & Sylvia 1992). The control group was fertilized with 10 mL of full‐strength Hoagland solution (Hoagland & Arnon 1938). Because the non‐inoculated control received full‐strength Hoagland solution while all inoculated treatments received half‐strength Hoagland solution, *ex vitro* comparisons reflect the combined effects of inoculation and nutrient regime and do not isolate an inoculation‐only effect. A nutrient‐matched non‐inoculated control (half‐strength Hoagland) was not included; therefore, causal attribution to inoculation alone is avoided in *ex vitro* interpretations. Survival rate was evaluated after 25 days, and phytotechnical variables were measured after 60 days under *ex vitro* conditions. At the end of the experiment, phytotechnical variables, phenols and flavonoids were evaluated.

### Bioactive compound extraction, quantification and data analysis

Hydro‐methanolic extracts were prepared using a methanol:water solution (80:20, v/v) acidified with 0.1% HCl. For each treatment, 0.2 g of dried leaf material was mixed with 10 mL of the extraction solution and agitated in the dark for 24 h. After this period, the extracts were centrifuged at 8550 × g for 15 min (Kasvi K14‐0815a), and the supernatants were used to quantify total phenolic content (TPC) and total flavonoid content (TFC) (Ariza *et al*. 2016; Ariza *et al*. 2018).

The TPC was evaluated by the Folin–Ciocalteu (FC) method with adaptations (Singleton & Rossi 1965). Briefly, 100 μL of hydro‐methanolic extract was mixed with 500 μL of 10% FC reagent and incubated for 3 min. Subsequently, 400 μL of CH_3_CO_2_Na (7 M) was added, and the solution was incubated at room temperature in the dark for 2 h. Absorbance was measured at 760 nm using a spectrophotometer (Thermo Scientific™ Multiskan™). The calibration curve was prepared with gallic acid standard solution (100 μg/mL), and results were expressed as mg GAE/g (milligrams of gallic acid equivalents per gram of dry weight) (Simirgiotis *et al*. 2008; Ariza *et al*. 2016; Ariza *et al*. 2018).

The TFC was quantified according to Ariza *et al*. (2016), with modifications. A volume of 250 μL of hydro‐methanolic extract was mixed with 1.25 mL of MilliQ water and 75 μL of 5% NaNO_2_ solution and incubated for 6 min. Then, 150 μL of 10% AlCl_3_·6H_2_O solution and 500 μL of 1 M NaOH were added, and the final volume was adjusted to 2.5 mL with MilliQ water. Absorbance was measured at 420 nm using a spectrophotometer (Thermo Scientific™ Multiskan™). The standard curve was prepared with quercetin standard solution (100 μg/mL), and results were expressed as mg QE/g (milligrams of quercetin equivalents per gram of dry weight) (Chang *et al*. 2002; Farajzadeh Memari‐Tabrizi *et al*. 2021).

The data obtained were first tested for normality using the Shapiro–Wilk test. Analysis of variance (ANOVA) was then performed, and means were compared using Tukey's test at a significance level of *P* < 0.05. Statistical analyses were performed in R 4.5.2 using the packages tidyverse and easyanova (Arnhold 2013; Wickham *et al*. 2019).

## RESULTS

### Bacterial diversity

At the phylum level, Actinomycetota and Bacillota were dominant across all samples. However, relative abundances shifted over successive generations. G0 displayed decreasing proportions of Pseudomonadota and Bacillota in comparison to the mother plant (Mother–PM), while the proportions of Actinomycetota increased. The abundance of minor phyla, such as Bacteroidota and Chloroflexota, declined rapidly to the point that they were not detected or did not reach 1% relative abundance to be considered separate taxa in earlier generations (Fig. 1A).

**Fig. 1:**
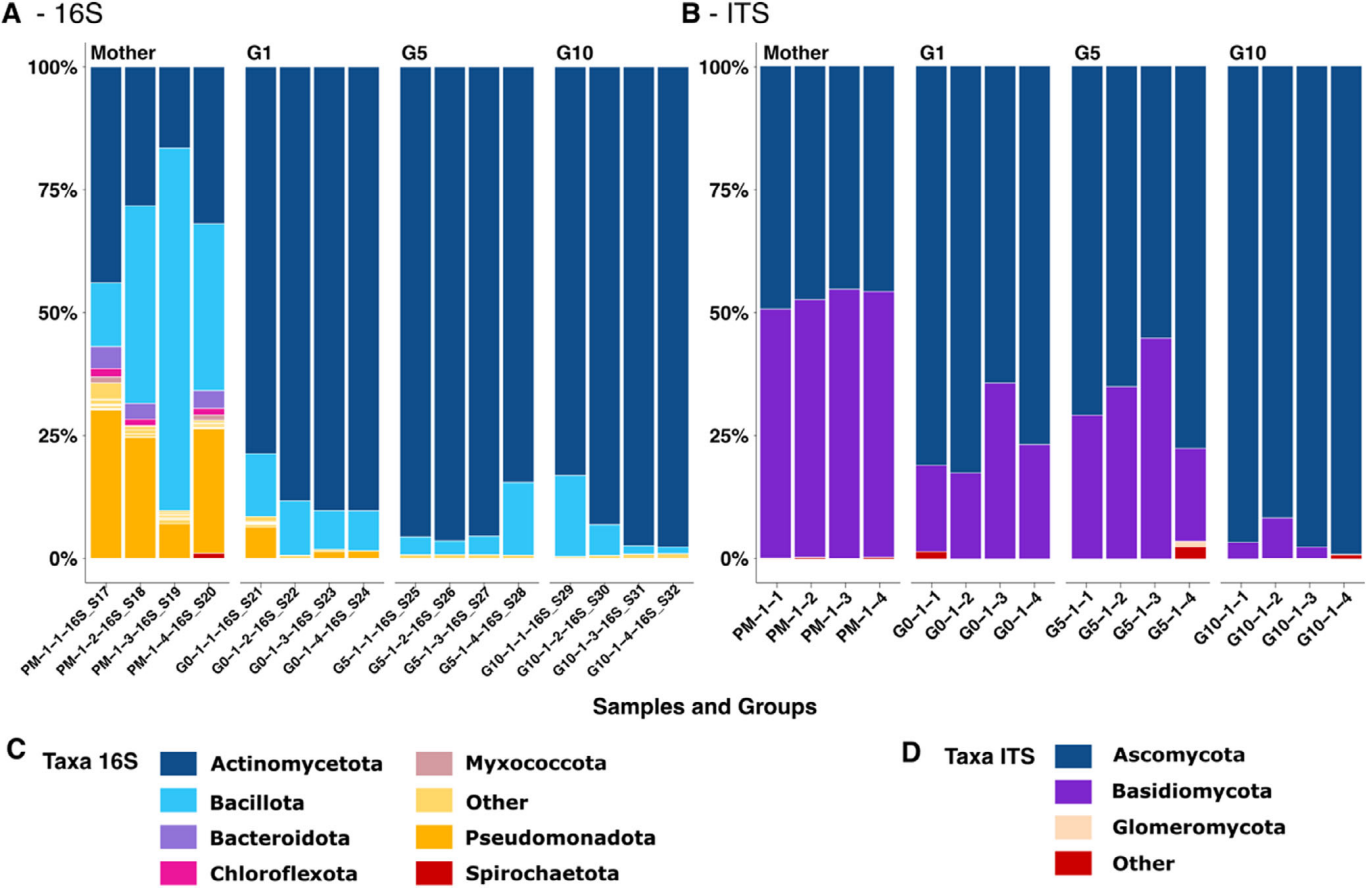
Relative abundance of (**A**) bacterial and (**B**) fungal phyla in endophytic communities of the strawberry cultivar *San Andreas*. Data are shown for the mother plant (Mother‐PM) and successive *in vitro* generations (G1, G5, and G10). Only phyla with relative abundance >1% are represented; taxa below this threshold were grouped as ‘Other’. Bars represent the proportional distribution of taxa within each sample, grouped according to treatment. Colour legends indicate the taxa identified by (**C**) 16S rRNA gene and (**D**) ITS sequencing.

The beta diversity analysis using Bray–Curtis‐based NMDS (Fig. 2A) showed distinct clustering of samples by treatment, with clear separation between the mother plant and the later generations (G0, G5 and G10; Stress = 0.036). A reduction in beta diversity from the mother plant to subsequent generations is also evident, as indicated by increased clustering within the same treatment. PERMANOVA confirmed that treatment significantly influenced the structure of bacterial communities (F = 23.37, *P* = 0.001).

**Fig. 2:**
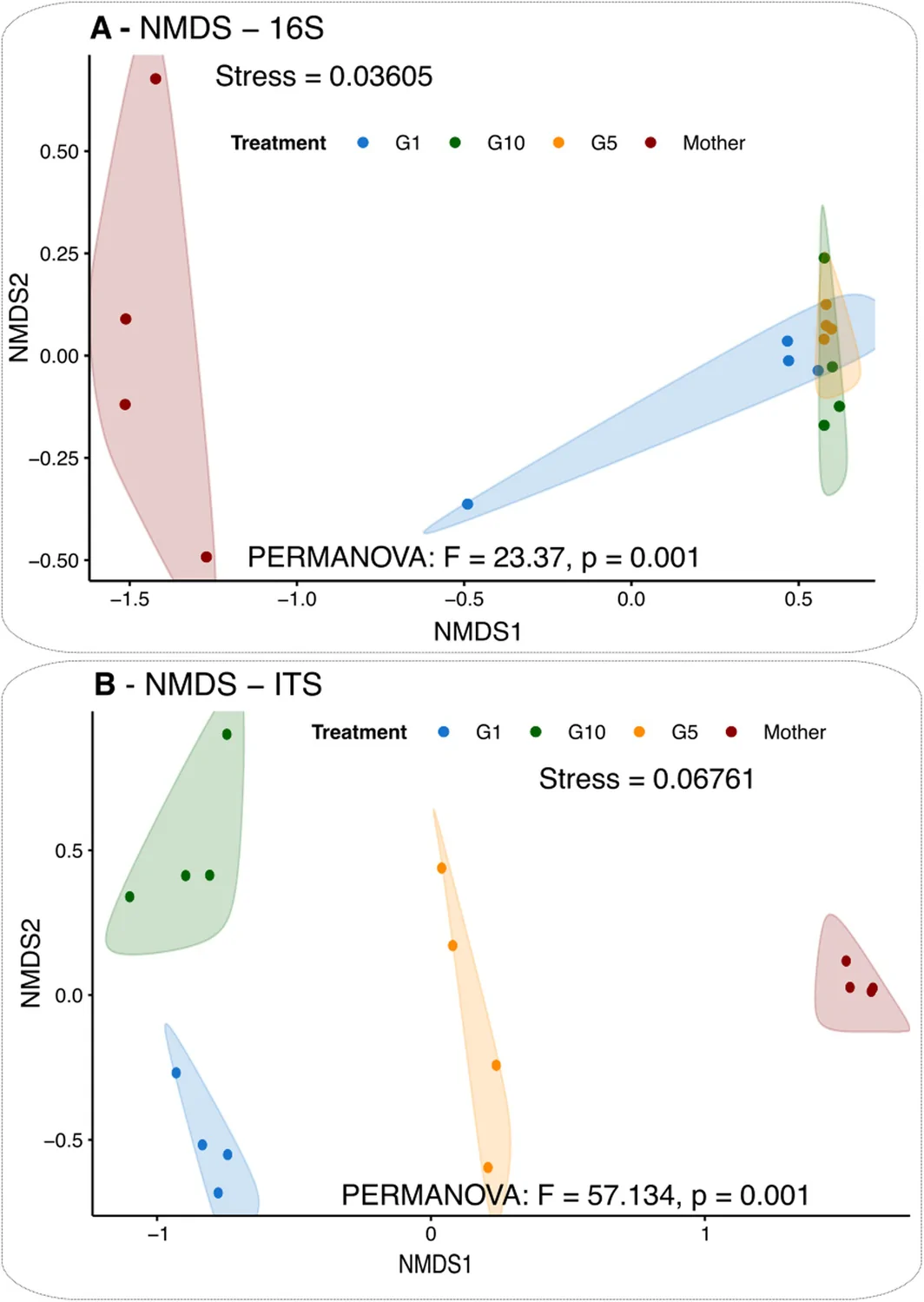
Non‐metric multidimensional scaling (NMDS) based on Bray–Curtis dissimilarity of (**A**) bacterial 16S rRNA and (**B**) fungal ITS amplicon data. Samples cluster by treatment (PM, G0, G5, G10), illustrating differences in endophytic bacterial community structure across strawberry micropropagation generations. Stress value and PERMANOVA results (F and *P*‐value) are indicated. Treatments are encircled to highlight group clustering.

A heatmap (Fig. 3A) of the top 20 most abundant genera revealed shifts in dominant genera along the subcultures. Genera such as *Novosphingobium*, *Rhizobium*, *Roseateles* and *Bradyrhizobium* were more abundant in the mother plant and early generations, whereas *Microbacterium* and *Nocardia* became more prominent in later passages (G5 and G10), highlighting a restructuring of the endophytic bacterial community associated with strawberry micropropagation.

**Fig. 3:**
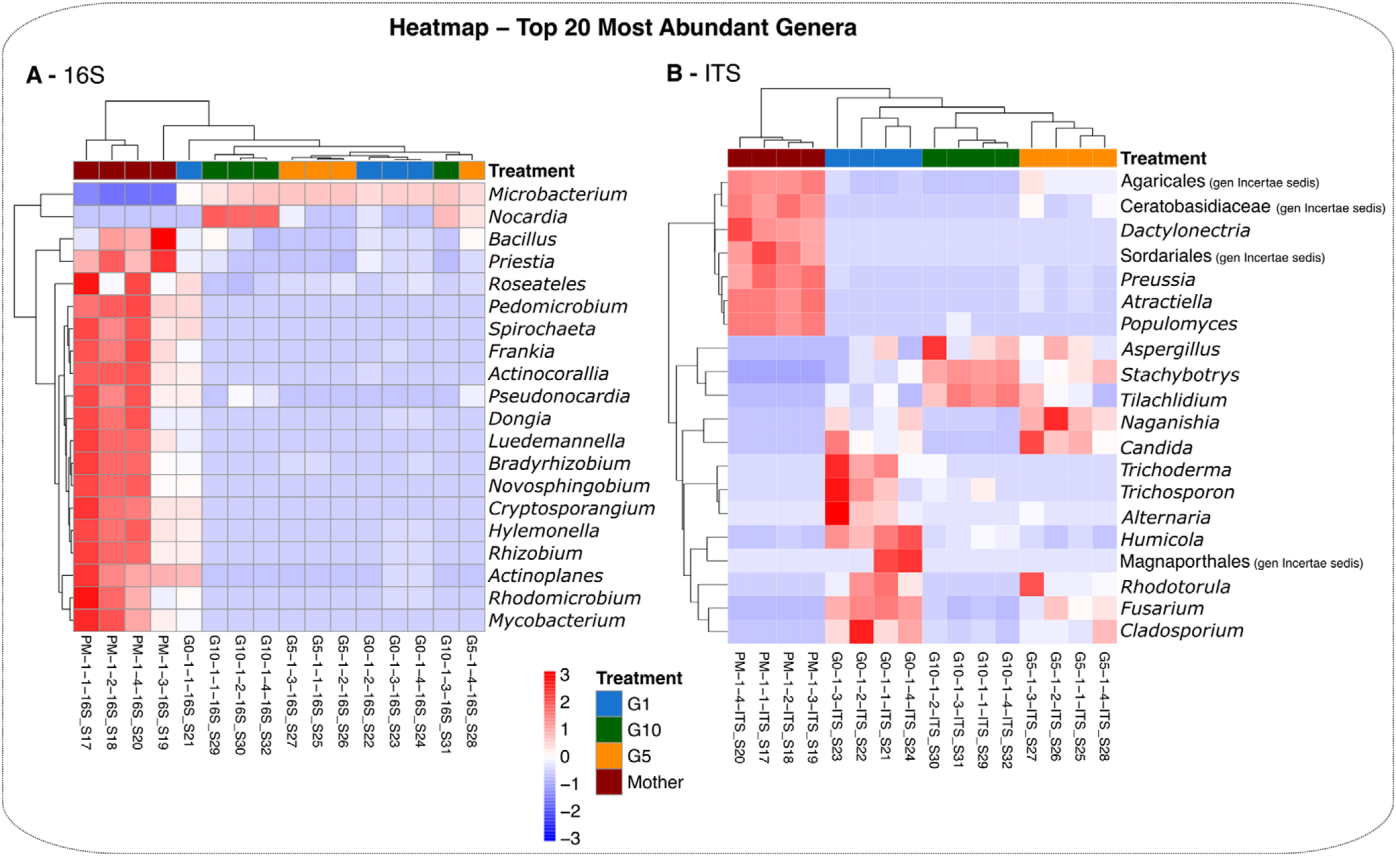
Heatmap showing the relative abundance of the 20 most prevalent (**A**) bacterial and (**B**) fungal genera detected in the endophytic microbiome of strawberry (*Fragaria × ananassa* cv. San Andreas). Treatments correspond to the mother plant (PM) and successive *in vitro* generations (G0, G5, G10). Clustering samples illustrates the differences in composition among treatments and clustering taxa indicates co‐occurrence.

Bacterial richness decreased from an average of 101.25 observed ASVs in the mother plant to 28.00 in G10. Likewise, Shannon diversity declined from 3.71 to 1.71 (all *P* < 0.001), indicating a marked simplification of the endophytic community (Fig. 4). Simpson's index also decreased from 0.95 in the mother plant to 0.78 in G10, while Pielou's evenness declined from 0.80 to 0.52. All comparisons between the mother plant and G1, G5 and G10 were significant (all *P* < 0.001).

**Fig. 4:**
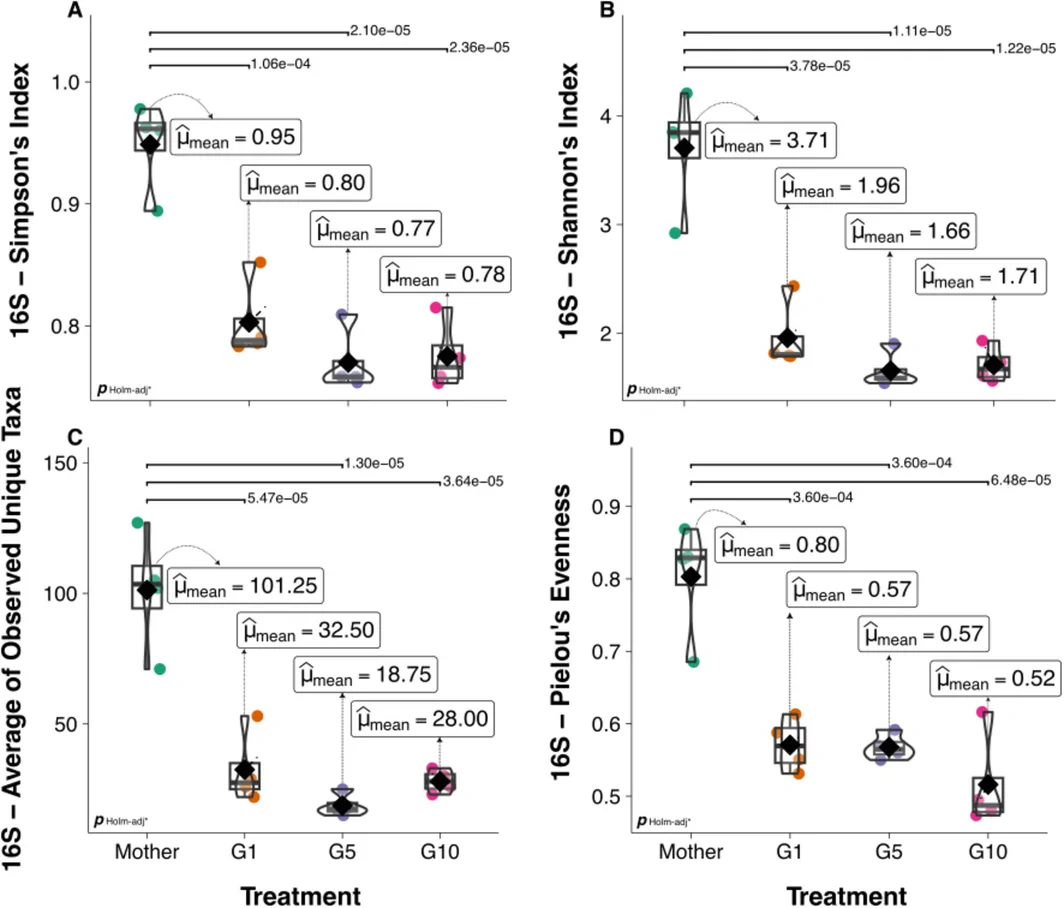
Alpha‐diversity of bacterial endophytic communities (16S rRNA gene) across micropropagation generations. (**A**) Simpson's index, (**B**) Shannon's index, (**C**) observed unique taxa (richness) and (**D**) Pielou's evenness for the Mother plant and *in vitro* generations (G1, G5, G10). Points represent individual samples, distributions are shown as violin plots with embedded boxplots, and black diamonds indicate group means. Horizontal brackets indicate significant pairwise comparisons (*t*‐test) with Holm‐adjusted *P* values shown above the brackets.

### Fungal diversity

The analysis of the fungal community revealed clear shifts in taxonomic composition and community structure across treatments. At the phylum level, the communities were dominated by Ascomycota and Basidiomycota, with minor contributions of Mucoromycota and unclassified fungi. Although all treatments showed similar dominant phyla, their relative abundances varied with the micropropagation stage and inoculation treatment (Fig. 1B).

Bray–Curtis‐based NMDS demonstrated a clear separation of fungal communities among treatments (PERMANOVA, *P* < 0.05). Samples clustered according to the generation and inoculation treatment, indicating that both plant propagation stage and microbial inoculation influenced the structure of the endophytic fungal community (Fig. 2B).

At a finer taxonomic resolution, the heatmap constructed at the genus level showed distinct community profiles among treatments (Fig. 3B). A limited number of genera dominated each treatment, while others were either treatment‐specific or present in low abundance. The heatmap highlighted shifts in dominant taxa across micropropagation generations and in response to different inoculation strategies, suggesting that the fungal microbiome is highly dynamic and responsive to experimental conditions.

Alpha‐diversity patterns for the fungal endophytic community are summarized in Fig. 5, and significance refers to the Holm‐adjusted pairwise *t*‐tests shown as brackets in the figure. Fungal alpha‐diversity did not show a uniform monotonic decline across all *in vitro* generations. Instead, Simpson's index was similar in G1 and the mother plant (0.84 in Mother vs. 0.91 in G1), then decreased in later generations (0.75 in G5 and 0.43 in G10), with significant (*P* < 0.05) contrasts indicated. Shannon's index followed the same overall shape, increasing from 2.21 (Mother) to 2.80 (G1), remaining similar in G5 (2.03) and dropping sharply in G10 (1.03). Richness (observed unique taxa) showed a modest increase from Mother (25.00) to G1 (27.50), then decreased in G5 (17.75) with partial recovery in G10 (22.25). Evenness increased from 0.69 (Mother) to 0.85 (G1), stayed similar in G5 (0.66) and declined strongly in G10 (0.36), consistent with greater dominance in the late‐generation fungal community, as reflected by the significant comparisons.

**Fig. 5:**
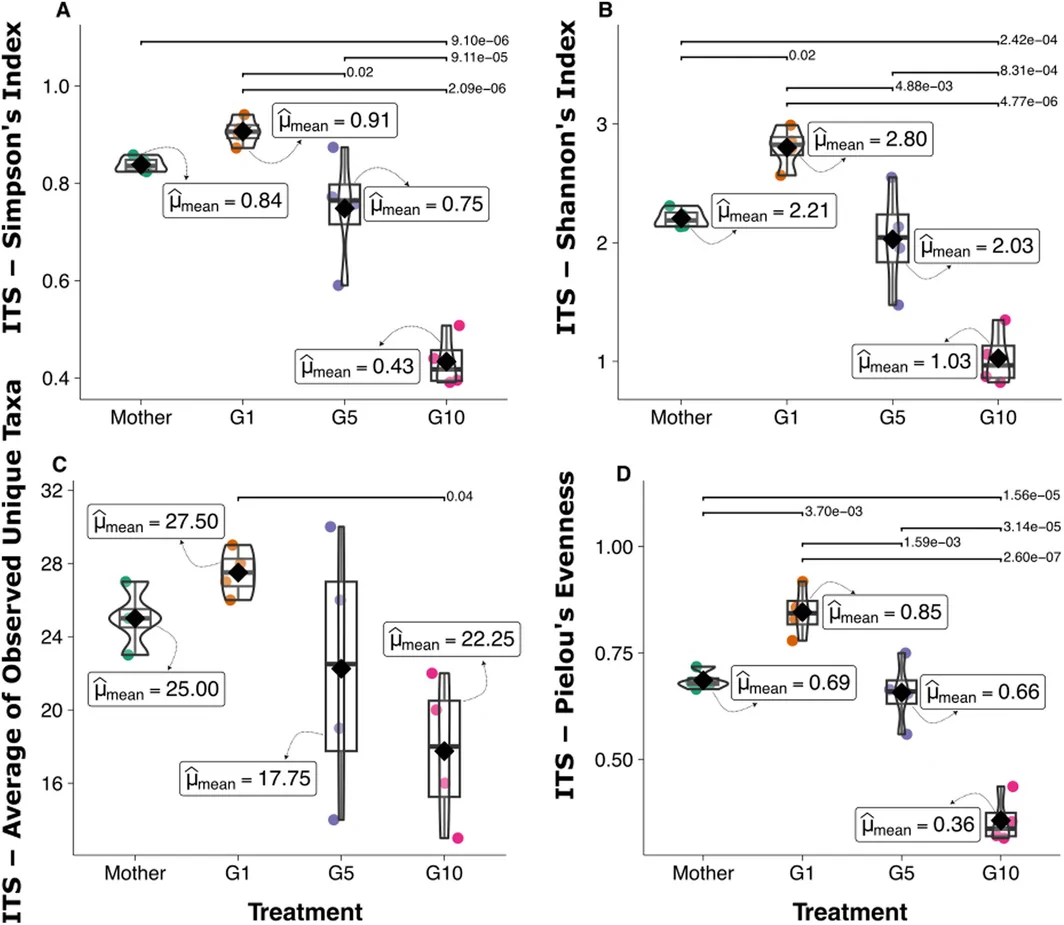
Alpha‐diversity of fungal endophytic communities (ITS rRNA) across micropropagation generations. (**A**) Simpson's index, (**B**) Shannon's index, (**C**) observed unique taxa (richness) and (**D**) Pielou's evenness for the Mother plant and *in vitro* generations (G1, G5, G10). Points represent individual samples, distributions are shown as violin plots with embedded boxplots, and black diamonds indicate group means. Horizontal brackets indicate significant pairwise comparisons (*t*‐test) with Holm‐adjusted *P* values shown above the brackets.

### Response of cultivar SA to individual biotization

The results of the individual biotization indicated that 30% of the tested strains positively influenced shoot length (Table 1, Section 1), shoot dry biomass (SDM), root dry biomass (RDM) and total dry biomass accumulation (TDM), with the strains *Azospirillum brasilense* Ab‐V5, *Azospirillum brasilense* Ab‐V6 and *Bacillus subtilis* CCMA 0401 showing the most pronounced effects.

In contrast, the strains *Rhizobium leucaneae* BR935, *Sinorhizobium fredii* BR921 and *Rhizobium tropici* CIAT 899 showed values similar to those of the control. Moreover, a negative effect on the development of microplants biotized with *Bacillus megaterium* CCMA 0004, *Bradyrhizobium japonicum* CCMA 0088 and *Sinorhizobium fredii* CCMA 0122 was observed after 30 days of *in vitro* micropropagation. The strain *Bacillus amyloliquefaciens* CCMA0112 was excluded from the experiment after 72 h of co‐cultivation because it exhibited extensive visible bacterial proliferation on both the culture medium and the surface of the microplants. This assessment was based on periodic qualitative visual observations during the experiment. Unlike the other strains, CCMA0112 rapidly colonized the entire culture medium and plant surface, resulting in microplant death within 72 h after inoculation.

### Quantification of phenols and flavonoids *in vitro*

The quantification of phenols and flavonoids revealed a similar accumulation pattern across treatments. The consortium Ab‐V6 + BR935 showed the highest concentrations of Total Phenolic Content (TPC, 15.20 mg GAE g.dw^−1^) and Total Flavonoid Content (TFC, 64.12 mg QE g.dw^−1^), representing increases of 136.8% and 112.5%, respectively, relative to the non‐inoculated control. This was followed by Ab‐V5 + 0401, Ab‐V6 + 0401 and Ab‐V5 for TPC, and by Ab‐V5 + 0401 and Ab‐V6 + 0401 for TFC. The remaining treatments showed values close to those of the control, with 6.42 mg GAE g.dw^−1^ for TPC and 30.17 mg QE g.dw^−1^ for TFC (Fig. 6A,B).

**Fig. 6:**
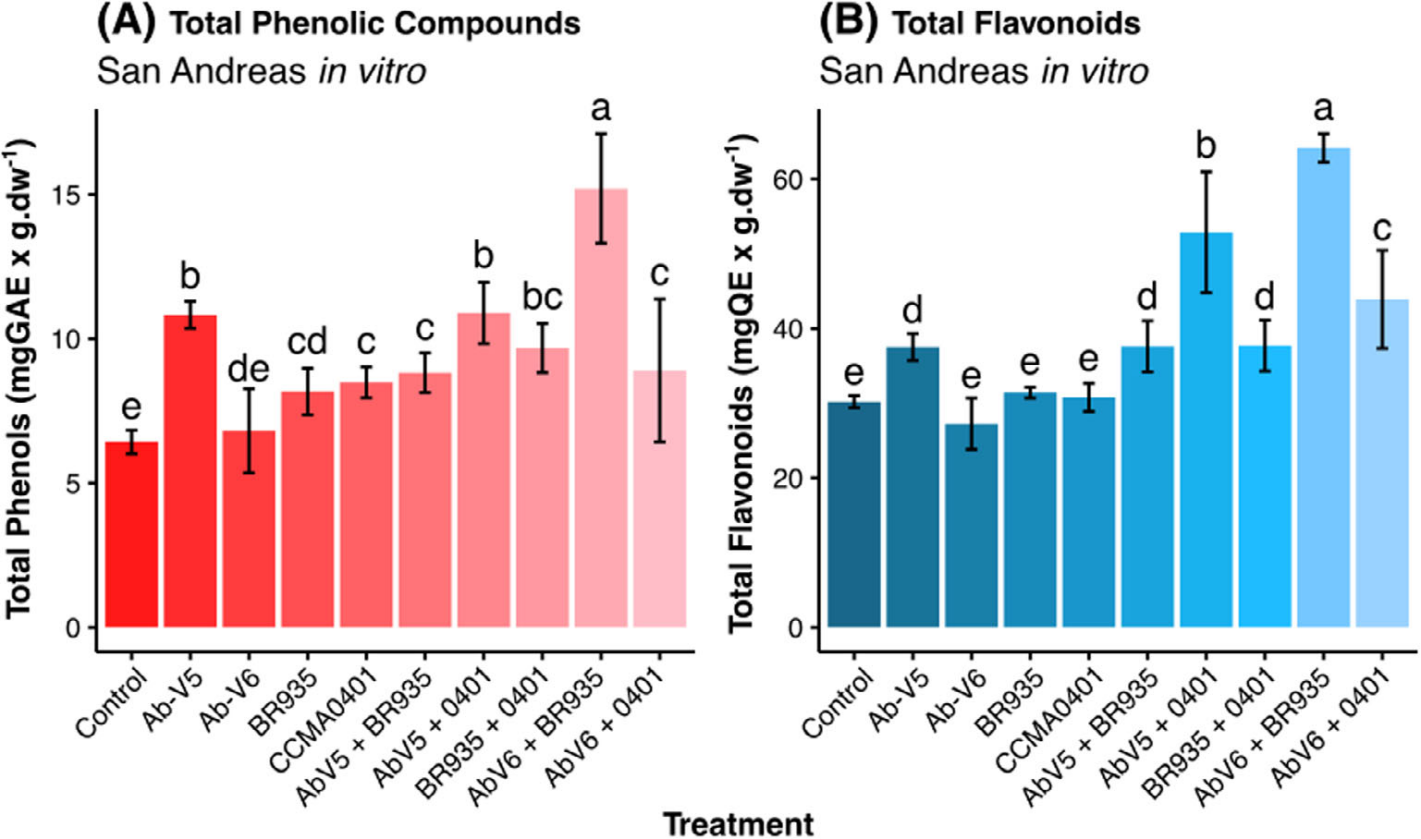
(**A**) Quantification of Total Phenolic Content (TPC) and (**B**) Total Flavonoid Content (TFC) in the *San Andreas* cultivar under *in vitro* conditions. Different letters indicate statistically significant differences (*P* < 0.05).

### Acclimatization of the San Andreas cultivar to *ex vitro* conditions

After transferring the plantlets from *in vitro* to *ex vitro* conditions, the survival rate was calculated. The biotized plantlets (Group A) showed that all four individual treatments, as well as the consortia Ab‐V5 + 0401 and BR935 + 0401, achieved 100% survival. Because the *ex vitro* control and inoculated treatments differed in Hoagland strength (full vs. half), the patterns reported here should be interpreted as combined inoculation plus nutrient‐regime effects rather than inoculation alone. The plantlets subjected to a second reinoculation demonstrated that treatments Ab‐V5, CCMA0401, Ab‐V5 + BR935, Ab‐V5 + 0401 and BR935 + 0401 also maintained 100% survival. In contrast, the remaining treatments exhibited survival rates close to or below that of the control, with the consortia containing strain Ab‐V6 showing only 50% survival, while the control treatment achieved 75% (Table S3).

### Phytotechnical variables under *ex vitro* conditions of the San Andreas cultivar

Under *ex vitro* conditions, significant differences among treatments were observed for several phytotechnical variables (Table 1). Although shoot length was not affected in biotized plants, treatments containing Ab‐V5, CCMA0401 and BR935 + 0401 generally promoted greater biomass accumulation, while the consortia Ab‐V5 + 0401 and BR935 + 0401 consistently improved root development. Following reinoculation, Ab‐V5 + 0401 and Ab‐V5 + BR935 showed the best overall performance, whereas consortia containing Ab‐V6 consistently exhibited the weakest responses (Table 1, Sections 3 and 4).

### Quantification of phenolic compounds under *ex vitro* conditions in the San Andreas cultivar

Under *ex vitro* conditions, biotized plants showed increased total phenolic content (TPC) in several inoculated treatments relative to the control (Fig. 7A). The consortium Ab‐V5 + BR935 exhibited the highest TPC, followed by treatments Ab‐V5, Ab‐V5 + 0401, Ab‐V6 and BR935 + 0401, whereas CCMA0401 showed the lowest accumulation among the inoculated treatments. Detailed values and statistical comparisons are presented in Fig. 7A.

**Fig. 7:**
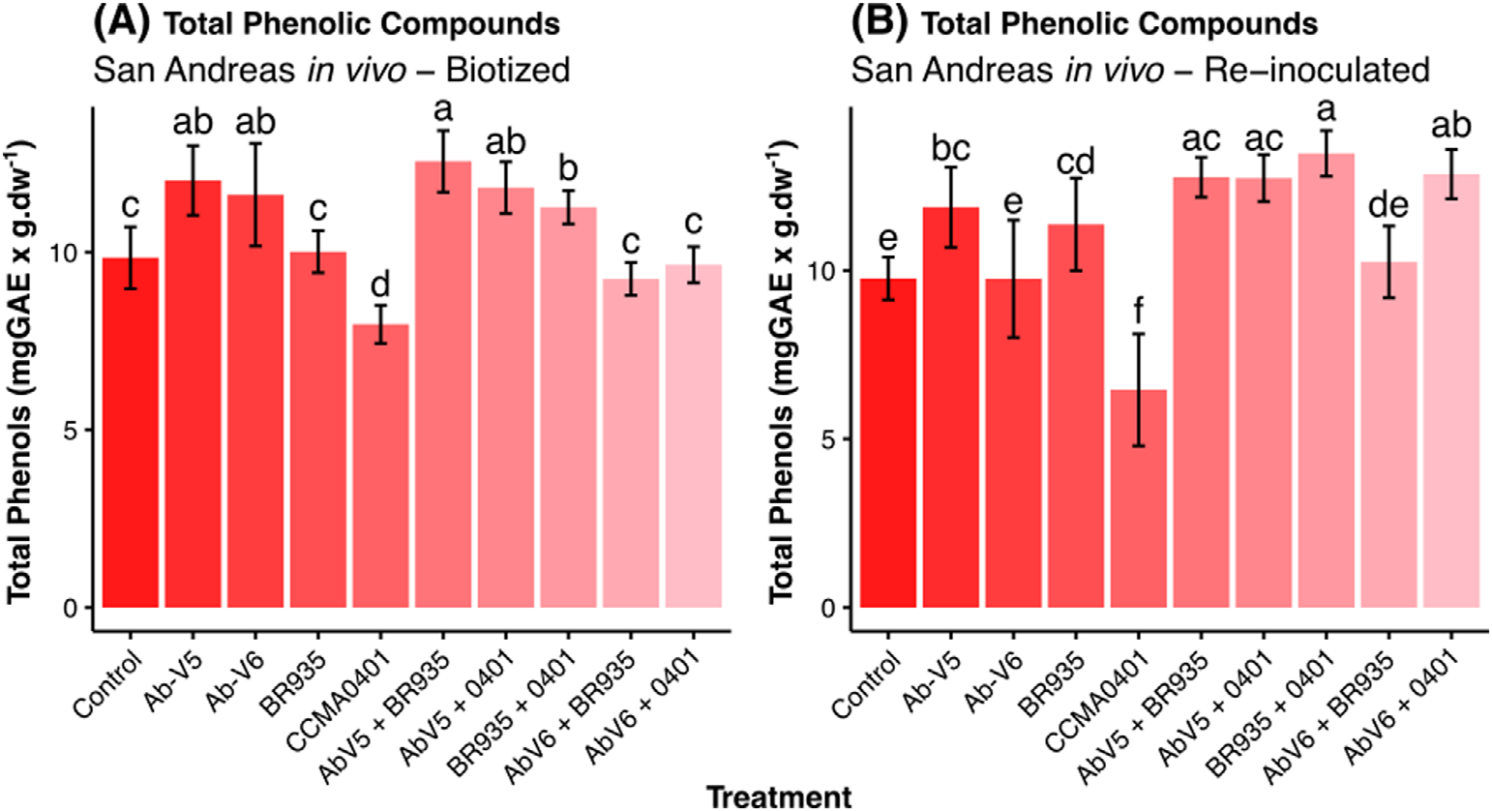
Total Phenolic Content (TPC) in *San Andreas* strawberry plants under *ex vitro* conditions. (**A**) Biotized; (**B**) Re‐inoculated. Different letters indicate statistically significant differences (*P* < 0.05).

Following reinoculation, TPC increased in several treatments (Fig. 7B). The consortium BR935 + 0401 produced the highest TPC, followed by Ab‐V6 + 0401, Ab‐V5 + BR935 and Ab‐V5 + 0401. The remaining treatments showed values similar to or lower than the control, with CCMA0401 again presenting the lowest TPC.

### Quantification of flavonoids under *ex vitro* conditions in the San Andreas cultivar

Under *ex vitro* conditions, flavonoid accumulation varied among inoculation treatments (Fig. 8). In biotized plants, the highest total flavonoid content (TFC) was observed for the consortia Ab‐V5 + 0401 and Ab‐V5 + BR935, whereas the remaining treatments were similar to or lower than the control (Fig. 8A). After reinoculation, Ab‐V5 + BR935 remained the best‐performing treatment, followed by Ab‐V5 + 0401, while the remaining treatments did not differ substantially from the control (Fig. 8B).

**Fig. 8:**
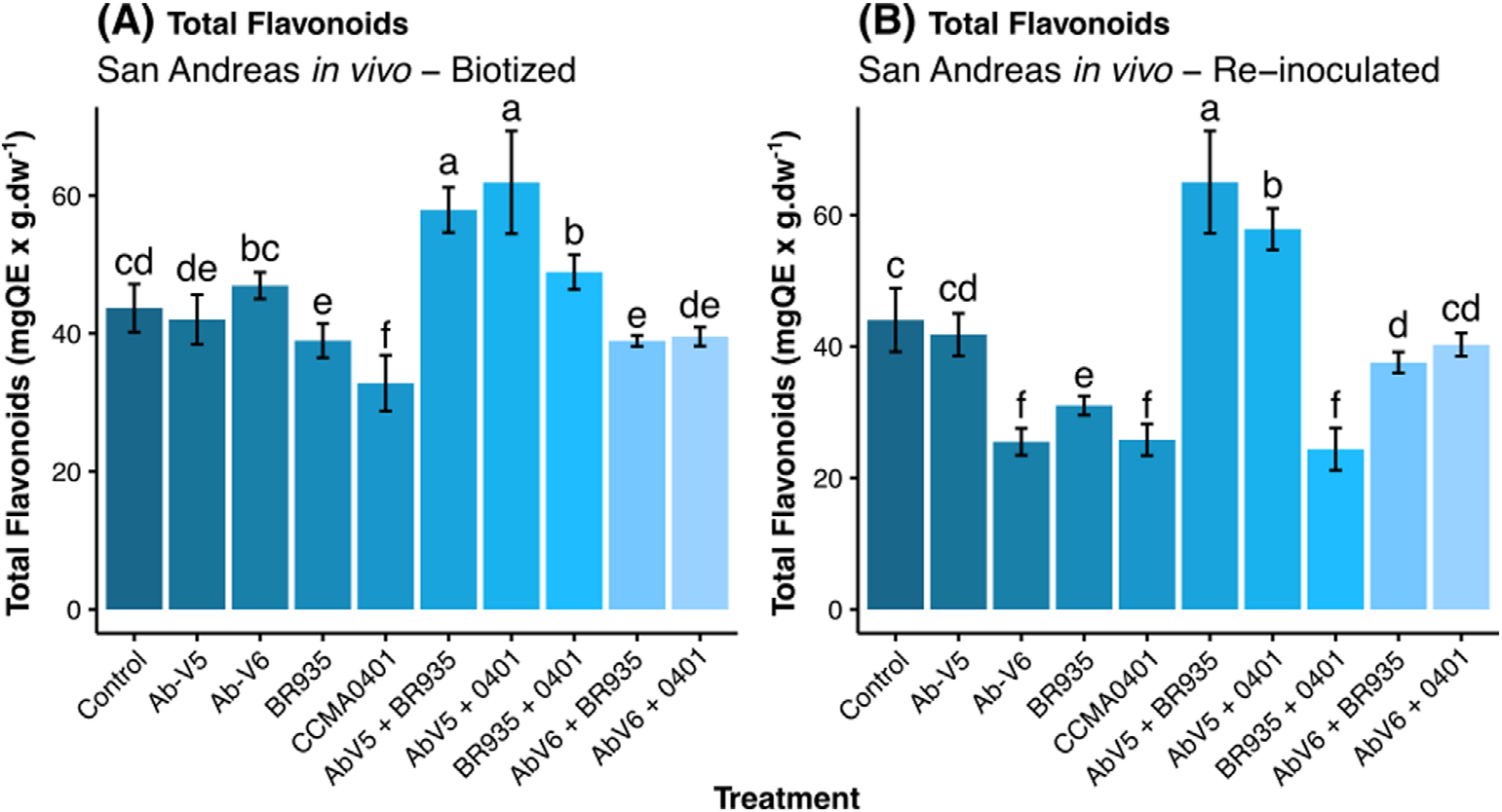
Total Flavonoid Content (TFC) in *San Andreas* strawberry plants under *ex vitro* conditions. (**A**) Biotized; (**B**) Re‐inoculated. Different letters indicate statistically significant differences (*P* < 0.05).

## DISCUSSION

### Endophytic plant microbiome in *in vitro* strawberry culture

The plant microbiome plays a crucial role in plant productivity and health, influencing growth, disease resistance and tolerance to biotic and abiotic stresses (Hardoim *et al*. 2015). In this context, endophytic microbiomes are particularly relevant, as sequencing technologies have enabled more detailed analysis of these communities (Jansson & Baker 2016), revealing important ecological and functional interactions with host plants.

Factors such as plant age, genotype and environmental conditions significantly influence endophytic microbial assembly. Colonization often occurs through microbial selection in the rhizosphere, mediated by plant genetic factors that facilitate microorganism entry into the roots (Haldar & Sengupta 2015; Knights *et al*. 2021). In this study, 16S rRNA gene sequencing revealed that the mother plant (PM), which was maintained *ex vitro*, exhibited greater bacterial richness and diversity than micropropagated generations (G0, G5, G10) cultured *in vitro* for 10 months. These *in vitro* generations showed greater similarity in bacterial composition, although slight variations were detected, particularly in G0. These findings align with previous reports showing that tissue culture systems usually exhibit reduced endophytic diversity compared to *ex vitro* plants, since *in vitro* cultures originate from single explants (here, stolons) and only a subset of endophytes survives the sterilization process prior to culture establishment (Quambusch *et al*. 2014; Orlikowska *et al*. 2016; Quambusch & Winkelmann 2018).

The endophytic microbiota of the mother plant comprised seven bacterial phyla, with *Firmicutes*, *Actinobacteriota* and *Proteobacteria* being dominant, and 17 genera were identified. The distribution in PM differed substantially from that *in vitro*. The *in vitro* cultures showed an increased relative abundance of *Actinobacteriota* (>0.8%) in all generations, followed by *Firmicutes*. At the same time, *Proteobacteria* appeared only in G0 and disappeared thereafter, suggesting a loss of this group during subculturing. This pattern is consistent with previous findings in *Arbutus unedo* L., where *Actinobacteriota* was predominant (48%), followed by *Proteobacteria* (43%), *Firmicutes* (6%) and *Bacteroidota* (3%) under *in vitro* conditions, with similar though variable compositions *ex vitro* (Martins *et al*. 2022).

In this study, *Microbacterium* was the most abundant genus across *in vitro* generations, followed by *Bacillus*, *Frankia*, *Hylemonella* and *Allorhizobium–Neorhizobium–Pararhizobium–Rhizobium*. In G5 and G10, diversity decreased drastically, leaving *Microbacterium* dominant. Comparable patterns were reported in *A. unedo*, where *Mycobacterium*, *Cutibacterium* and *Corynebacterium* were the most abundant *Actinobacteriota*, while *Pseudomonas* and *Serratia* dominated the *Proteobacteria* fraction (Martins *et al*. 2022).

The predominance of *Microbacterium in vitro* aligns with studies identifying *Microbacterium zeae* sp. nov. as an endophyte of maize stems (Gao *et al*. 2017) and with other *Microbacterium* species that exhibit phosphate‐solubilizing and auxin‐producing capabilities (Walitang *et al*. 2017). Research on field‐grown strawberries also showed that *Actinobacteriota*, *Alphaproteobacteria*, *Gammaproteobacteria*, *Deltaproteobacteria* and *Bacteroidia* composed nearly 80% of the endophytic community, with genotype and tissue type strongly influencing community structure (Sangiorgio *et al*. 2022). Our results indicate that *ex vitro* plants retain greater microbial diversity, whereas prolonged *in vitro* subculturing leads to microbiome simplification and selection for resilient taxa, such as *Actinobacteriota*.

The marked restructuring of the endophytic microbiome across successive *in vitro* generations is likely driven by several ecological filters. Surface sterilization reduces microbial diversity by eliminating epiphytes, while only some endophytes survive in plant tissues. Repeated subculturing under controlled conditions, including artificial media, humidity, gas exchange, immigration and no natural microbial inputs, imposes continuous selective pressure. Microorganisms better adapted to the *in vitro* environment dominate, while less competitive or host‐dependent taxa are lost. Similar processes have been observed in other micropropagated plant species (Xiang *et al*. 2023; Vinskienė *et al*. 2025).

Studies have further shown that endophytes such as *Paenibacillus lautus* can produce plant hormones and secondary metabolites, thereby promoting biomass accumulation and antioxidant enzyme activity (Ptak *et al*. 2022). Such evidence supports the role of endophytes in sustainable agriculture. However, some latent microorganisms may emerge during *in vitro* micropropagation (Abreu‐Tarazi *et al*. 2009; Almeida *et al*. 2009; Esposito‐Polesi *et al*. 2015; Esposito‐Polesi *et al*. 2016). No visible endophytic manifestation was observed in this study over 10 months of subculturing *San Andreas* plantlets.

For the bacterial (16S) communities, the consistent reductions in richness, diversity and evenness across all *in vitro* generations indicate a strong simplification of the bacterial community relative to the mother plant. Because Shannon and Simpson decreased together with richness and evenness, the shift is not only a loss of taxa but also an increase in dominance, consistent with a selective bottleneck during micropropagation and maintenance under *in vitro* conditions. The persistence of low alpha‐diversity from G1 through G10 suggests that most of the community restructuring occurs early and then stabilizes, implying that subsequent subculturing maintains a comparatively constrained community rather than progressively eroding diversity across generations (Morris *et al*. 2014; Tamang *et al*. 2023).

In contrast to the bacterial pattern of immediate and persistent alpha‐diversity loss relative to the mother plant, the fungal (ITS) community shows an early maintenance in diversity and evenness in G1, followed by a pronounced simplification in later generations. The combination of decreased Shannon and Simpson indices, together with the substantial drop in evenness in G10, indicates that the late‐generation fungal community is not only less diverse but also more dominated by a smaller subset of taxa. This supports a model where early *in vitro* transfer can transiently favour a broader or more evenly distributed fungal assemblage (for example, through altered competitive hierarchies immediately after transfer) but continued subculturing and sustained *in vitro* selective pressures ultimately impose a bottleneck that drives dominance and reduced community complexity, most evident in G10 (Morris *et al*. 2014; Newcombe *et al*. 2018; Sharon *et al*. 2024).

Overall, the *ex vitro* mother plant harboured a richer and more complex endophytic microbiome, whereas prolonged *in vitro* subculturing progressively selected a reduced subset of microorganisms adapted to tissue culture conditions. Such microbiome simplification may reduce the functional diversity available to the host, potentially affecting nutrient acquisition, stress tolerance and acclimatization capacity. Therefore, strategies aimed at preserving or re‐establishing beneficial microbial communities, such as targeted biotization, may contribute to improving the performance of micropropagated plants.

### Response of the San Andreas cultivar to individual and consortium biotization

The biotization of *in vitro* plantlets offers a promising strategy for investigating the physiological and metabolic responses of plant explants to co‐cultivation with plant growth‐promoting bacteria (PGPB). This process enables the selection of beneficial strains and consortia that enhance plant growth and metabolism. In this study, individual biotization of *San Andreas* with ten PGPB strains identified *Azospirillum brasilense* Ab‐V5, *A. brasilense* Ab‐V6 and *Bacillus subtilis* CCMA 0401 as the most effective in promoting biomass accumulation. *Rhizobium leucaneae* BR935, which showed growth similar to the control, was also used in consortia.

The combinations Ab‐V6 + 0401, Ab‐V5 + 0401 and Ab‐V5 + BR935 were the most effective in promoting plant growth under *in vitro* conditions. Regarding phenolic and flavonoid accumulation, the consortium Ab‐V6 + BR935 showed the highest values, even without a significant increase in biomass, suggesting that PGPB can influence secondary metabolism and induce biochemical changes during plant‐microbe interactions (Chamam *et al*. 2013; Miotto‐Vilanova *et al*. 2019; Mhlongo *et al*. 2020).

The increase in phenolic and flavonoid contents may reflect the activation of plant defence and secondary metabolic pathways following bacterial colonization. Beneficial microorganisms are known to stimulate induced systemic resistance and modulate signalling pathways involving jasmonic acid, salicylic acid and ethylene, leading to enhanced activity of the phenylpropanoid pathway and increased synthesis of antioxidant compounds. Such metabolic responses may improve plant fitness during acclimatization by increasing tolerance to oxidative and environmental stresses (Rahman *et al*. 2018; Morais *et al*. 2019; Gupta *et al*. 2021).

The growth‐promoting responses observed in the present study are likely the result of multiple complementary mechanisms rather than a single bacterial trait (Bashan & de‐Bashan 2010). Plant growth‐promoting bacteria can enhance plant development through biological nitrogen fixation, phosphate solubilization, phytohormone production, modulation of ethylene levels *via* ACC deaminase, siderophore production and improved nutrient acquisition. Together, these mechanisms promote root development, increase nutrient uptake efficiency and improve biomass accumulation, particularly under the restrictive conditions imposed by *in vitro* culture (Buttrós *et al*. 2022; Ali *et al*. 2024; Buttrós *et al*. 2025).

Some bacterial strains negatively affected strawberry microplant development, but the effects varied. *Bacillus amyloliquefaciens* CCMA0112 overproliferated on the medium and plant surface, causing microplant death within 72 h. In contrast, *Bradyrhizobium japonicum* CCMA0088, *Sinorhizobium fredii* CCMA0122, and Bacillus megaterium CCMA0004 did not stimulate growth despite no excessive bacterial proliferation, suggesting strain compatibility rather than bacterial overgrowth. These contrasting outcomes highlight the complexity of plant‐bacterium interactions *in vitro*, influenced by bacterial strain‐plant genotype compatibility, inoculum density, metabolism and tissue culture conditions (Fouda *et al*. 2019; del Rosario Espinoza‐Mellado *et al*. 2021; Fouda *et al*. 2021). Since a single inoculum concentration was used for all strains, optimizing inoculum density for individual microorganisms was beyond the scope of the present study and warrants future investigation.

Although bacterial colonization in the San Andreas cultivar was not quantitatively evaluated, this study aimed to establish an *in vitro* biotization protocol and identify plant growth‐promoting strains. Future studies should combine microbiological, molecular and microscopic approaches to quantify bacterial colonization and relate it to physiological responses after biotization (Orlikowska *et al*. 2016; Quambusch & Winkelmann 2018).

Previous studies have reported the success of biotization with *Methylobacterium* sp. L10 and *Stenotrophomonas* sp. L20 in *Jacaranda mimosifolia*, increasing multiplication rates and rooting percentages, achieving up to 95% survival after *ex vitro* acclimatization (Salotti *et al*. 2023). Similarly, inoculation of strawberries with Pedobacter sp. significantly increased leaf phenolic and flavonoid content, supporting its potential as a biofertilizer that strengthens plant defences and enhances fruit quality (Morais *et al*. 2019).

It is important to note that the bacterial strains evaluated in this study were not selected to replace specific taxa lost during successive *in vitro* generations. Instead, they were chosen based on their previously characterized plant growth‐promoting traits to evaluate whether beneficial microbial functions could improve plant performance despite the observed microbiome simplification.

### Response of the San Andreas cultivar to *ex vitro* conditions

Under *ex vitro* conditions, biotization with *A. brasilense* Ab‐V5, both individually and in consortia, significantly increased survival rates, while Ab‐V6 consortia showed weaker growth‐promoting responses. Plantlets treated with Ab‐V5, CCMA 0401 and BR935 + 0401 accumulated more dry biomass, and reinoculation with Ab‐V5, Ab‐V5 + 0401 or Ab‐V5 + BR935 further enhanced this effect. Phenolic and flavonoid contents were also modulated by PGPB presence.

The Ab‐V5 strain is a well‐established biofertilizer known for nitrogen fixation, phosphate solubilization and phytohormone production (Andrade *et al*. 2019; Pedraza *et al*. 2020). Biochemical characterization confirmed its ability to fix nitrogen, solubilize P, K and Zn, and produce IAA, gibberellins, salicylic acid and hydrolytic enzymes, including proteases. The superior performance of Ab‐V5 observed both *in vitro* and *ex vitro* is therefore likely associated with the combined action of these plant growth‐promoting mechanisms rather than a single physiological process.

Similarly, *Bacillus* species, renowned for their robustness and PGP traits, can colonize diverse environments through hormone production, nutrient solubilization and extracellular enzyme activity (Soni & Keharia 2021). The *B. subtilis* CCMA 0401 strain exhibited nitrogen fixation, P, K and Zn solubilization, production of amylases, cellulases, proteases, lipases and chitinases, as well as synthesis of gibberellins, salicylic acid, ACC deaminase, siderophores, HCN and moderate biofilm formation, attributes that likely contributed to plant acclimatization. Other reports have also documented the use of *Phyllobacterium* and *Rhizobium* in strawberry growth promotion (Flores‐Félix *et al*. 2018).

Although the Ab‐V6 + 0401 consortium was beneficial *in vitro*, it did not significantly promote *ex vitro* growth, likely due to microbial competition, environmental stress or differential metabolite accumulation under soil conditions. The reduced performance of Ab‐V6‐based consortia may be linked to their rapid growth compared to Ab‐V5, potentially altering consortium balance.

In *ex vitro* acclimatization, several inoculated treatments showed higher survival and/or growth metrics than the non‐inoculated control. However, because the control received full‐strength Hoagland while the inoculated treatments received half‐strength Hoagland, these differences cannot be attributed uniquely to inoculation. They may also reflect nutrient‐regime and stress‐physiology effects.

Overall, this study highlights the potential of both individual and consortium‐based PGPB inoculation strategies to improve the adaptation and performance of micropropagated *San Andreas* strawberry plants under *ex vitro* conditions, representing a promising biotechnological approach for sustainable tissue culture systems.

## CONCLUSION

Successive *in vitro* subculturing of strawberry plants was associated with a simplification of the bacterial endophytic microbiome, characterized by reduced diversity and a community increasingly dominated by Actinobacteriota and Firmicutes. In contrast, the *ex vitro* mother plants harboured a more diverse and taxonomically complex endophytic community. Biotization identified the strains Ab‐V5 and CCMA0401 and the consortium BR935 + 0401 as promising candidates for improving biomass accumulation, while Ab‐V5, Ab‐V5 + 0401 and BR935 + 0401 showed favourable responses during reinoculation under *ex vitro* conditions. Collectively, these findings suggest that inoculation with plant growth‐promoting bacteria may represent a useful strategy for improving the performance and acclimatization of micropropagated strawberry plants, potentially compensating for the functional consequences of microbiome simplification. These results warrant further investigation into microbiome‐assisted approaches for tissue culture systems.

## AUTHOR CONTRIBUTIONS

AMGS: Conceptualization; methodology; investigation; formal analysis; data curation; visualization; writing – original draft; writing – review and editing. EdCJ: Conceptualization; methodology; validation; supervision; writing – review and editing. YCM: Methodology; investigation; formal analysis; data curation; writing – review and editing. VHB: Methodology; formal analysis; visualization; software; writing – original draft; writing – review and editing. LQC: Investigation; methodology; data curation. MP: Supervision; project administration; funding acquisition. JD: Supervision; project administration; funding acquisition; writing – review and editing.

## Supporting information


**Table S1:** Primers for 16S and ITS rRNA sequencing.
**Table S2:** Bacterial strains used as single strains and consortia for in vitro biotization assay.
**Table S3:** Survival percentage of San Andreas strawberry microplants under ex vitro conditions.

## Data Availability

The data that supports the findings of this study are available in the Supporting Information – S1 of this article.
